# Erratum

**DOI:** 10.1080/13814788.2017.1420600

**Published:** 2017-12-22

Bordado Sköld M, Aabenhus R, Guassora AD, Mäkelä M. Antibiotic treatment failure when consulting patients with respiratory tract infections in general practice. A qualitative study to explore Danish general practitioners’ perspectives. Eur J Gen Pract. 2017;23:121-128.

https://doi.org/10.1080/13814788.2017.1305105

Papadakaki M, Lionis C, Saridaki A, Dowrick C, de Brún T, O’Reilly-de Brún M, O’Donnell CA, Burns N, van Weel-Baumgarten E, van den Muijsenbergh M, Spiegel W, MacFarlane A. Exploring barriers to primary care for migrants in Greece in times of austerity: Perspectives of service providers. Eur J Gen Pract. 2017;23:129-135.

https://doi.org/10.1080/13814788.2017.1307336

Martín-Lesende I, Orruño E, Mateos M, Recalde E, Asua J, Reviriego E, Bayón JC. Telemonitoring in-home complex chronic patients from primary care in routine clinical practice: Impact on healthcare resources use. Eur J Gen Pract. 2017;23:136-143.

https://doi.org/10.1080/13814788.2017.1306516

European General Practice Research Network (EGPRN): Abstracts from the EGPRN meeting in Leipzig, Germany, 12–16 October 2016. Theme: ‘General practice/family medicine in a changing world’. Eur J Gen Pract. 2017;23:144-155.

http://dx.doi.org/10.1080/13814788.2017.1300653

Pype P, Mertens F, Belche J, Duchesnes C, Kohn L, Sercu M, Deveugele M. Experiences of hospital-based multidisciplinary team meetings in oncology: An interview study among participating general practitioners. Eur J Gen Pract. 2017;23:156-164.

https://doi.org/10.1080/13814788.2017.1323081

Popa SG, Moţa M, Mihălţan FD, Popa A, Munteanu I, Moţa E, Serafinceanu C, Guja C, Hâncu N, Catrinoiu D, Lichiardopol R, Bala C, Mihai B, Radulian G, Roman G, Timar R. Associations of smoking with cardiometabolic profile and renal function in a Romanian population-based sample from the PREDATORR cross-sectional study. Eur J Gen Pract. 2017;23:165-171.

https://doi.org/10.1080/13814788.2017.1324844

Iatraki E, Simos PG, Bertsias A, Duijker G, Zaganas I, Tziraki C, Vgontzas AN, Lionis C, on behalf of the THALIS Primary Health Care Research Team/Network. Cognitive screening tools for primary care settings: examining the ‘Test Your Memory’ and ‘General Practitioner assessment of Cognition’ tools in a rural aging population in Greece. Eur J Gen Pract. 2017;23:172-179.

https://doi.org/10.1080/13814788.2017.1324845

Agenda. Eur J Gen Pract. 2017;23:180.

http://dx.doi.org/10.1080/13814788.2017.1310518

Zurmati AK, De Sutter A, Decat P. Primary healthcare for refugees: The Ponton experience. Eur J Gen Pract. 2017;23:181-182.

https://doi.org/10.1080/13814788.2017.1331011

Tudrej BV, Heintz A-L, Rehman MB, Marcelli D, Ingrand P, Binder P. Even if they are not aware of it, general practitioners improve well-being in their adolescent patients. Eur J Gen Pract. 2017;23:183-190.

https://doi.org/10.1080/13814788.2017.1346077

Raft CF, Bjerrum L, Arpi M, Jarløv JO, Jensen JN. Delayed antibiotic prescription for upper respiratory tract infections in children under primary care: Physicians’ views. Eur J Gen Pract. 2017;23:191-196.

https://doi.org/10.1080/13814788.2017.1347628

Dézsi CA, Dézsi BB, Dézsi AD. Management of dental patients receiving antiplatelet therapy or chronic oral anticoagulation: A review of the latest evidence. Eur J Gen Pract. 2017;23:197-202.

https://doi.org/10.1080/13814788.2017.1350645

Lionis C, Midlöv P. Prevention in the elderly: A necessary priority for general practitioners. Eur J Gen Pract. 2017;23:203-208.

https://doi.org/10.1080/13814788.2017.1350646

Subra J, Chicoulaa B, Stillmunkès A, Mesthé P, Oustric S, Rougé Bugat M-E. Reliability and validity of the script concordance test for postgraduate students of general practice. Eur J Gen Pract. 2017;23:209-214.

https://doi.org/10.1080/13814788.2017.1358709

When the articles listed above were originally published online, the page numbering was incorrect. This has now been corrected in both online and print versions.

Taylor and Francis apologizes for these errors.

